# Classification and prediction of toxicity of chemicals using an automated phenotypic profiling of *Caenorhabditis elegans*

**DOI:** 10.1186/s40360-018-0208-3

**Published:** 2018-04-18

**Authors:** Shan Gao, Weiyang Chen, Yingxin Zeng, Haiming Jing, Nan Zhang, Matthew Flavel, Markandeya Jois, Jing-Dong J. Han, Bo Xian, Guojun Li

**Affiliations:** 1 Beijing Key Laboratory of Diagnostic and Traceability Technologies for Food Poisoning, Beijing Center for Disease Prevention and Control/Beijing Center of Preventive Medicine Research, Beijing, 100013 China; 2 Key Laboratory of Computational Biology, CAS Center for Excellence in Molecular Cell Science, Collaborative Innovation Center for Genetics and Developmental Biology, Chinese Academy of Sciences-Max Planck Partner Institute for Computational Biology, Shanghai Institutes for Biological Sciences, Chinese Academy of Sciences, 320 Yue Yang Road, Shanghai, 200031 China; 3 College of Information, Qilu University of Technology (Shandong Academy of Sciences), Jinan, 250353 China; 4 School of Life Sciences, La Trobe University, Bundoora, Vic 3083 Australia; 5 Beijing Key Laboratory of Environmental Toxicology, School of Public Health, Capital Medical University, Beijing, 100069 China

**Keywords:** *C. elegans*, Chemicals, Toxicity, Image analysis, Phenotype

## Abstract

**Background:**

Traditional toxicological studies have relied heavily on various animal models to understand the effect of various compounds in a biological context. Considering the great cost, complexity and time involved in experiments using higher order organisms. Researchers have been exploring alternative models that avoid these disadvantages. One example of such a model is the nematode *Caenorhabditis elegans*. There are some advantages of *C. elegans*, such as small size, short life cycle, well defined genome, ease of maintenance and efficient reproduction.

**Methods:**

As these benefits allow large scale studies to be initiated with relative ease, the problem of how to efficiently capture, organize and analyze the resulting large volumes of data must be addressed. We have developed a new method for quantitative screening of chemicals using *C. elegans.* 33 features were identified for each chemical treatment.

**Results:**

The compounds with different toxicities were shown to alter the phenotypes of *C. elegans* in distinct and detectable patterns. We found that phenotypic profiling revealed conserved functions to classify and predict the toxicity of different chemicals.

**Conclusions:**

Our results demonstrate the power of phenotypic profiling in *C. elegans* under different chemical environments.

**Electronic supplementary material:**

The online version of this article (10.1186/s40360-018-0208-3) contains supplementary material, which is available to authorized users.

## Background

With the global development of science and technology, new chemicals are continuously synthesized and used in industrial production and human life. The need to assess the toxic effects of these chemicals on the environment, in addition to understanding the potential health impacts of these chemicals is an urgent and ongoing issue. The scientific community must take responsibility for this task and continue to re-evaluate the best methods for measuring toxicity. Assessment of the potential toxicity of chemicals is crucial for the determination of safe levels of exposure to humans. Routine methods to evaluate potential toxicity of a vast array of chemicals on humans have employed rodent animal models. Whilst very useful, this strategy is time consuming and very expensive, in addition to the ethical concerns it raises. The importance of these studies which have incorporated mammalian model animals cannot be denied as they have been integral in establishing the Globally Harmonized System (GHS) of Classification and Labeling of Chemicals [1], in addition to countless breakthrough studies. However, if the field of toxicology is to progress, it will first need to develop more progressive models of testing for toxicity.

Toxicity tests often utilize large numbers of rodents, and is extremely expensive and time consuming, which has resulted in a backlog of over 10,000 novel compounds that are urgently awaiting further testing [2]. Current in vivo rodent models of toxicity can no longer meet the increasing requirements of stringent toxicological evaluation. Clearly, there is a critical need to develop more rapid and efficient techniques of toxicity screening. In vivo studies in non-rodent model organisms provide a novel platform for screening a large number of chemicals for toxicity [3–10].

In recent years, the nematode *Caenorhabditis elegans* has been utilized in toxicity testing as an alternative in vivo animal model. *C. elegans* is a free-living nematode that lives mainly in the liquid phase of soils. Since it was first developed for use as a model animal in 1965 [11], it has been widely employed in various fields such as developmental biology, aging, neurobiology, etc. [12, 13]. Its widespread application is mainly due to a range of advantageous traits. Some key examples include a relatively short life and reproduction cycle, plus an extremely small body size which contribute to making it very easy and cheap to maintain large populations in the laboratory. A trait particularly useful for toxicological studies is the ease with which a vast array of *C. elegans* phenotypes are able to be quantified before and after the administration of a treatment. This includes the ability to define changes to both internal and external phenotypes due to the transparent nature of the body. In addition*, C. elegans* is the first multicellular organism whose genome has been fully sequenced. Within this genome of 20,000 genes, about 40% of *C. elegans* genes are homologous with mammals [14]. Of this homology, many integral metabolic pathways share distinct similarity between worm and mammal. From a toxicological perspective this makes *C. elegans* a very interesting prospect as it allows the metabolic effects of compounds to be incorporated into studies. This was demonstrated by our previous study [15] that showed the correlation of *C. elegans* LC50s (24 h) to rat LD50s is similar to the relationship between mouse and rat LD50s (*r* = 0.879), and was stronger than the correlation of NHK cell median inhibition concentrations (IC50)s vs. rat LD50s (*r* = 0.844). These results indicate that *C. elegans* may be a valuable model for predicting chemicals’ acute toxicity in rodents.

In recent years, several studies have tried to incorporate worms into toxicological screening studies [2, 4, 6]. These studies demonstrated that *C. elegans* is able to be utilized as a model to test the effects of chemicals toxicities in vivo. However, these studies relied on the COPAS system, which is a very expensive piece of equipment that is difficult for researchers to access. In addition, COPAS system only provides a limited range of data on features such as worm length, width and fluorescence intensity. This method of processing does not meet the throughput demands of the modern chemical research community.

The need to develop and improve automated strategies to gather phenotypic data is essential to furthering our understanding of biological functions in relation to chemical compounds. Recently, there are some interesting methods emerging that can be used to quantify phenotypes observed in *C. elegans* [16, 17]. These are predominantly designed for analyzing the images from nematode growth medium (NGM) agar plate experiments and determining the expressed phenotypes. However, they mainly focus on the quantification of only four phenotypes and the images are required to be extremely uniform in nature for the image processing to be completed accurately. This requirement for strict uniformity becomes an issue when the range of conditions that *C. elegans* are housed in is considered. For example, in a situation such as a 384 well plate experiment, and the well boundary and some uneven illumination problems would impact the results of current automated methods.

A cheap, fast and quantitative method for toxicity screening in *C. elegans* would benefit the field immensely. Here we report a method that combines a liquid toxic culturing system utilizing a 384-well plate with an automated microscope stage for image acquisition. We have utilized this method to classify automatically the toxicity of 19 compounds, under multiple concentrations according to specifically identified features of worms’ phenotypes. By analyzing our defined phenotypes of worms exposed to chemicals at different concentrations, we found that phenotype profiling revealed stable functions to classify and predict the toxicity of different chemicals.

## Results

### Phenotypes profiling

**Fig. 1:**
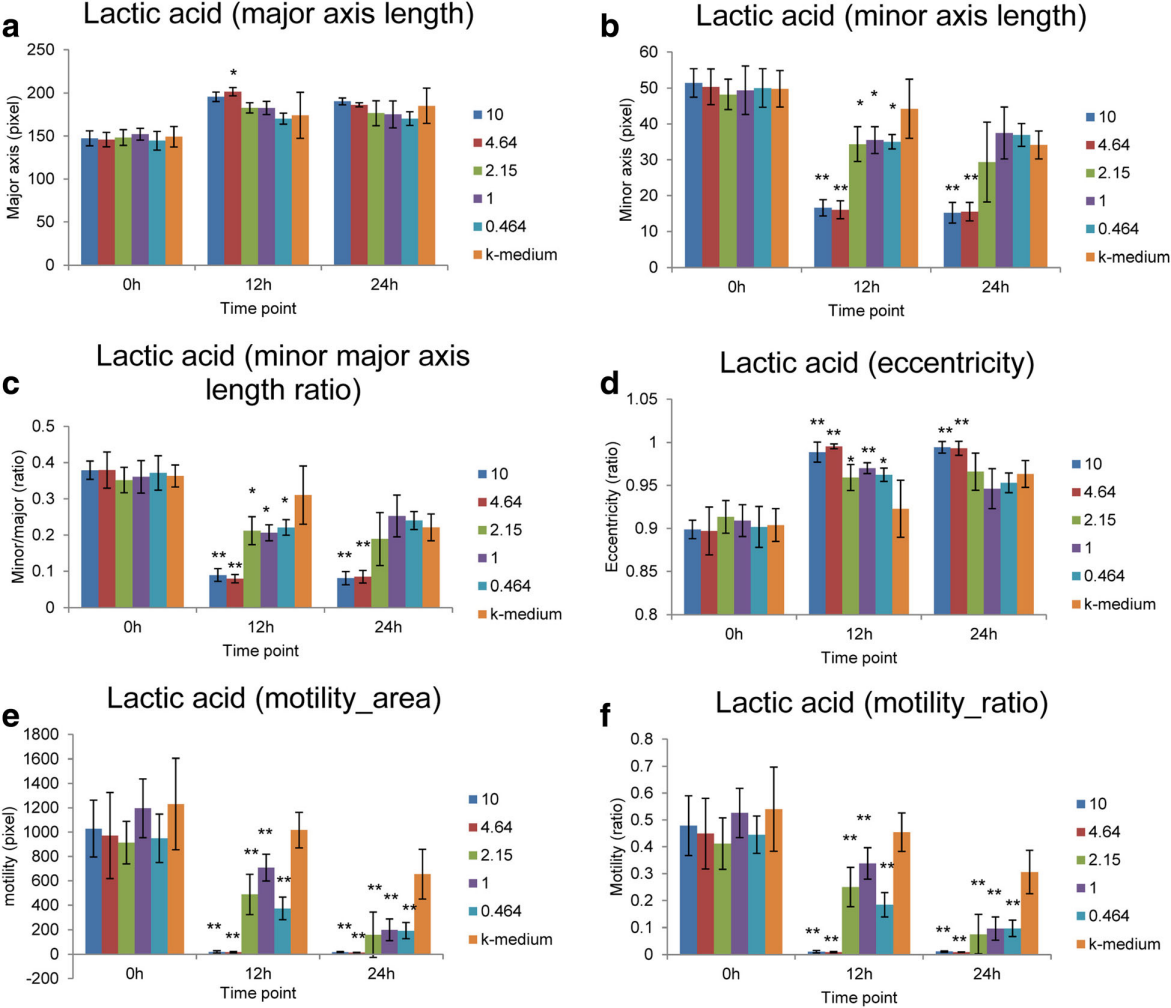
Phenotypes of lactic acid under different concentrations. (**a**) Major axis length. (**b**) Minor axis length. (**c**) Minor major axis length ratio. (**d**) Eccentricity. (**e**) Motility (the moved area). (**f**) Motility (the moved area/worm size). * and ** denote unpaired two-sided Student’s *t*-Test *p*-value < 0.05 and 0.01, respectively. Bar plots shows the average quantification for each phenotype on single worms. Error bars denote +/− standard deviation (SD). Concentration unit: mg/ml

From the large amount of image data processed, 33 distinct phenotypes were quantified (methods) across the 19 chemical compounds and at each of the 3 time points (0, 12 and 24 h), respectively. All compounds tested were shown to influence phenotype at certain time points and concentrations. In the interest of keeping this article succinct, of the 19 compounds tested, only the phenotype profile observed during treatment with lactic acid has been presented graphically to demonstrate the accuracy and depth of data collection that is possible using our methodology (Fig. 1). Extra examples of phenotypic profile data have been included in the Additional file 1: Figures S1 and S2. These supplementary figures show similar patterns to lactic acid, description of results will be limited to those observed in lactic acid.

The worms died quickly and became straighter and less curved as the chemical concentration increased. There were no significant differences between treatments and control (k-medium) for all phenotypes at the initial time point of the experiment (0 h). After 12 h of exposure to a given chemical dosage, the worms showed a range of phenotypic variations among different concentrations and control groups. For example, the major axis length (see phenotypes description) increased in all experiments. This was demonstrated by a gradient trend from higher to lower chemical concentrations (Fig. 1a). The trend is more significant in minor axis length. At 12 h, the Student’s *t*-Test shows that all experiments with 5 different concentrations are significantly different from control (Fig. 1b, *p*-value = 0.02121, 0.02889, 0.01905, 1.919e-05 and 2.501e-05 for 5 concentrations from lower to higher concentrations). Our findings for the 24 h 50% lethal concentration (LC50 as determined by previous studies [15]) there is already a clear distinction between higher and lower concentrations at the time point of 12 h. Two concentrations (10 mg/ml and 4.64 mg/ml) which are both higher than the LC50, show lower level of minor axis length compared with the other three concentrations (Fig. 1b).

The ratio between single worms’ minor and major axis length shows similar patterns as minor axis length (Fig. 1c), the higher values indicate the worms would be observed as more bent. The eccentricity shows the opposite pattern, because lower eccentricity values indicate the worm would appear more bent (Fig. 1d).

In terms of quantified worm motility, both the worm moved area and the moved ratio (moved area/worm size) show similar patterns (Fig. 1e, f). At the 0 h time point, there were no significant differences among worms across all experiments. As time passed, the motility of worms in control k-medium conditions showed a stable decrease. At 12 h, all the treatments showed very significant differences compared with control at that time point. We found there are drastic differences in motility for the concentrations higher or lower than the LC50 (2.79 mg/ml for Lactic Acid, see Additional file 1: Table S1.) at 24 h. Two concentrations (10 mg/ml and 4.64 mg/ml) which are both higher than the LC50 for Lactic Acid show lower levels of motility compared with the other three concentrations. This suggests that worms died quickly and became less motile as the chemical concentration increased.

These results indicate that the phenotypes we quantified are valid potential markers for chemical toxicity identification in *C. elegans*.

### Phenotypes classification

**Fig. 2:**
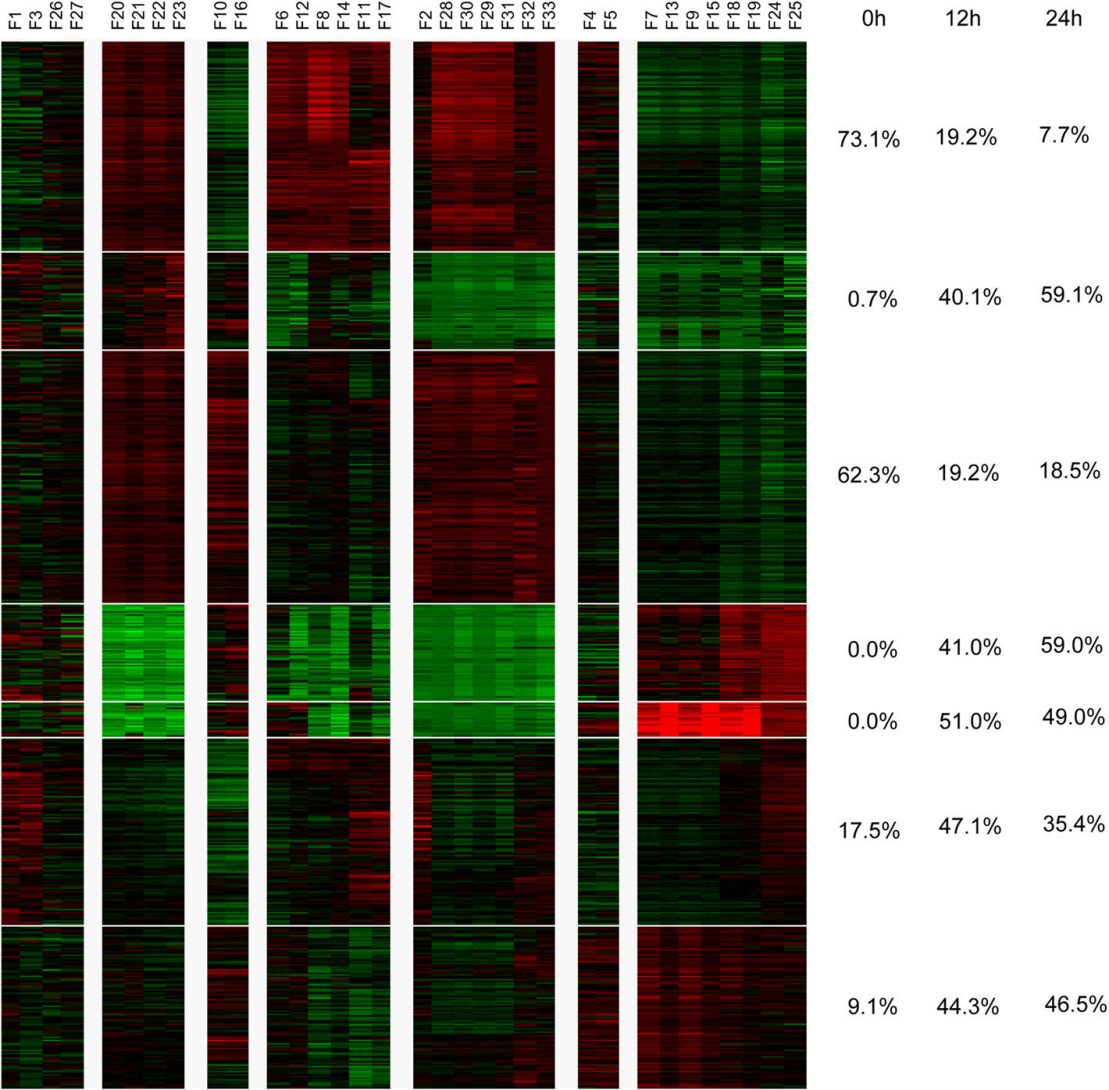
Cluster on all phenotypes of all experiments (3 time points). Each column is one feature (See Methods for a detailed name and description of each feature), each row is an experiment. The proportions of each time point’s experiments (in each sub-cluster) are listed in the right of heat map

To visualize the various phenotypes and treatments’ relationships among each other, all features were z-score normalized within each feature. This allowed clustering across different phenotypes and experiments using the BIC-SK algorithm [18], which is an adaptive cluster method. This method can automatically determine the optimal number of samples and feature clusters based on the Bayesian Information Criterion. With this approach, we were able to detect and visualize the various phenotypes at the same time. We find that all 33 quantified phenotypes were able to be classified accurately into distinct groupings. This grouping was also possible at each of the time points measured (0 h, 12 h and 24 h) (Fig. 2).

We found that at 0 h experiments have different patterns compared with later time points. The features which are based on all single worms or only live single worms respectively, show similar patterns compared with other features. It indicates that it is more similar within the same measurement than different measurements.

Among these features worm length, perimeter, major axis length and eccentricity have similar patterns. Survival rate and worm motility are clustered together. Most of the 0 h data shows higher survival rate and motility compared to later time points. This is in addition to later time points showing greater length and perimeter measurements compared to 0 h observations.

**Fig. 3:**
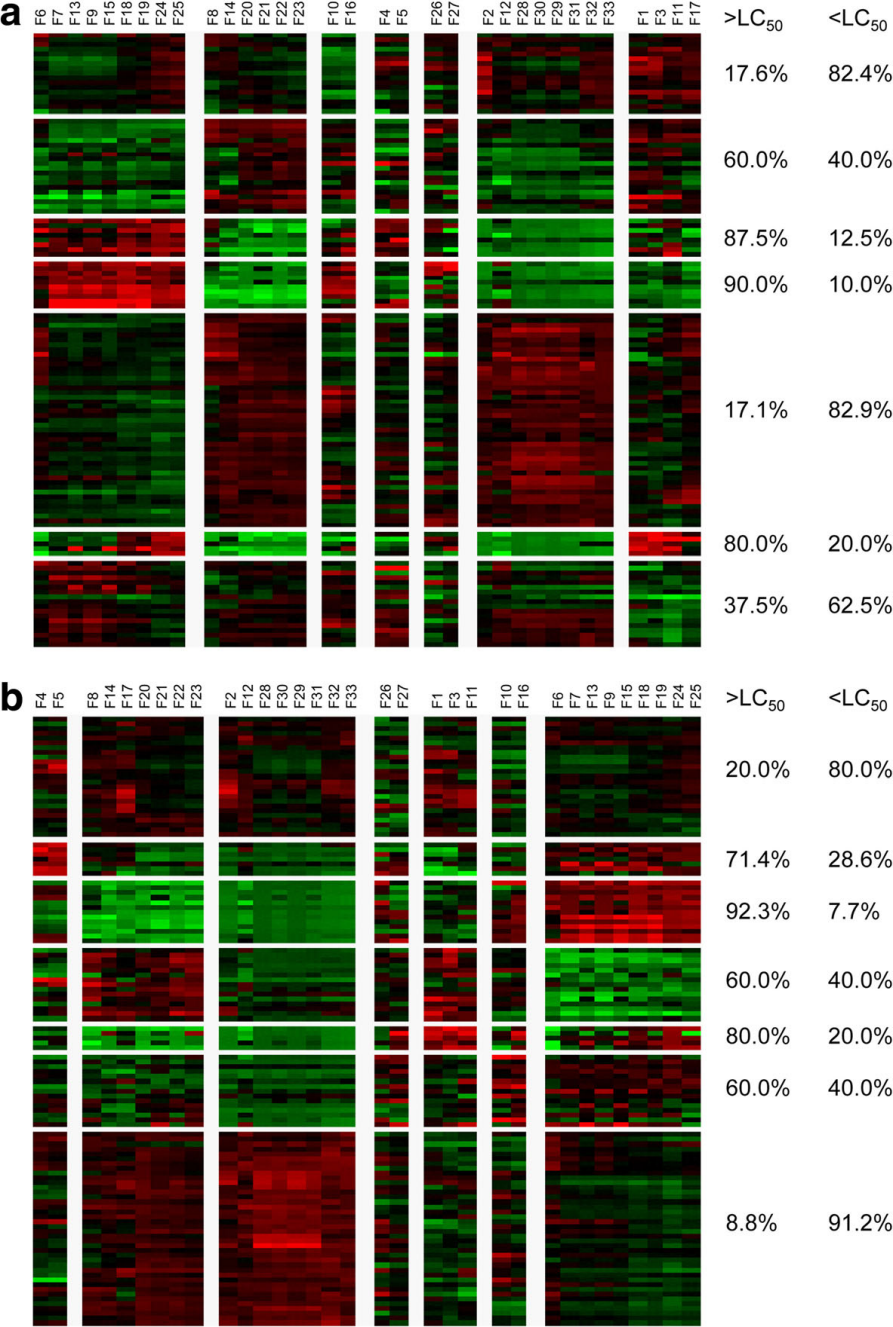
**a** Cluster on the mean profile of 8 repeats, one time point (12 h). **b** Cluster on the mean profile of 8 repeats, one time point (24 h). Each column is one feature (we show the detailed name of each feature in Methods). The proportions of different concentrations’ experiments (in each sub-cluster) are listed in the right of heat map. LC_50_ is 50% lethal concentration

In the cluster of one time point (12 h, Fig. 3a), we can see that higher concentration experiments (and therefore a higher degree of toxicity) have lower survival rate and motility than lower concentration experiments (lower toxicity). In the higher concentration experiments lower levels of minor axis length, minor/major axis length, higher levels of major axis length and eccentricity are all observed. In contrast, the lower concentration experiments show higher levels of minor axis length and minor/major axis length, lower levels of major axis length and eccentricity.

The 24-h results (Fig. 3b) are comparable to the 12-h results. It was observed that higher concentration experiments (high toxicity) have lower survival rate and motility than lower concentration experiments (lower toxicity).

From the clustering results it appears worms died quicker, were less motile, straightened and therefore showed less body curvature as the concentration increased.

### The main trends under these phenotypes

To detect whether time, concentration and toxicity associated principle components underlie these quantified phenotypes; we performed Principle Component Analysis (PCA). The advantage of using PCA is that it can summarize all phenotypes to several main components.

**Fig. 4:**
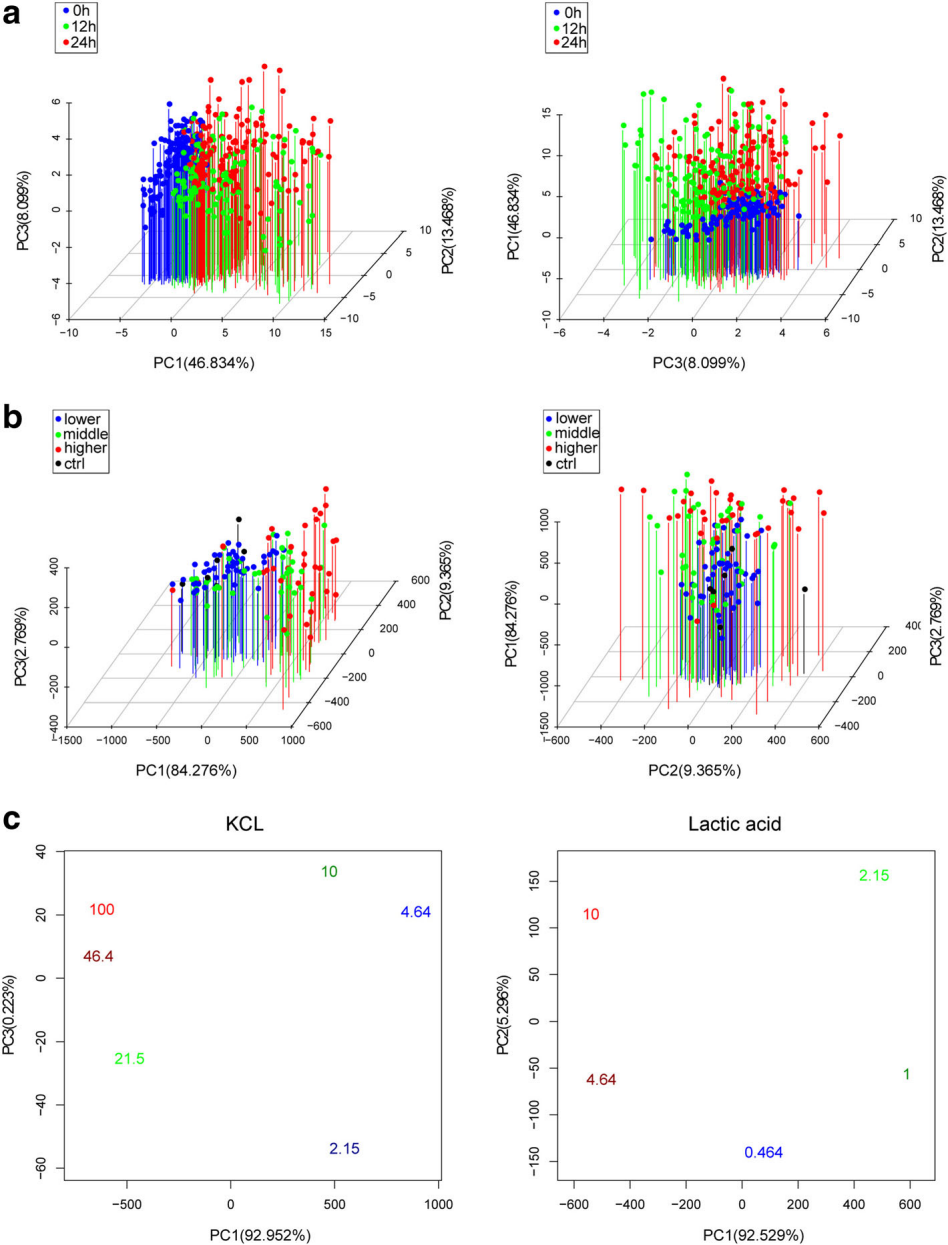
**a** PCA on the mean profile of 8 repeats (3 time points). **b** PCA on the mean profile of 8 repeats (12 h). **c** PCA on the mean profile of single chemical compound (KCL and Lactic acid of 12 h), the value with different color is the concentration of different experiment

Firstly, PCA results on the mean profile of 8 repeats (3 time points, Fig. 4a) show some main components to distinguish different time points. Three different colors represent the experiments under different time points. We found that the first component can distinguish 0 h time point from the other two time points. The increase of the third component shows that there is one change trend from 12-h to 24-h.

The PCA on the phenotypes observed at the 12-h time point and the concentration of each experiment was ranked according to relative order for each chemical compound (Fig. 4b). We found the experiments with lower concentrations were similar to the control, whilst being drastically different from the middle and higher concentration experiments for the first main component. In the second main component, most of higher concentration experiments show marked differences from lower and middle concentrations.

From the single chemicals PCA result (Fig. 4c), we found the first main component shows considerable differences between higher and lower concentrations. Concentrations can be separated into two parts from left to right. Each part also has some difference from upper to lower shown by the third main component.

### Toxicity prediction

In general, 50% lethal dosage (LD50) and 50% lethal time (LT50) are used to describe the chemicals’ toxicity in rodents (rat/mouse) model in vivo. The correlation of *C. elegans* LC50s (24 h) vs. rat LD50s is equal to the correlation of mouse vs. rat LD50s (*r* = 0.879), and is stronger than the correlation of NHK cell median inhibition concentrations (IC50)s vs. rat LD50s (*r* = 0.844), which indicate that *C. elegans* may be a valuable model for predicting chemicals’ acute toxicity in rodents [15].

The 24-h 50% lethal concentration is derived from previous independent experiment results [15]. Using the methods already described we found that the quantified phenotypes correlate closely with chemical concentration. Then we used these quantified phenotypes to predict if the concentration is under/above the 24 h 50% lethal concentration (LC50). For each chemical, we classified different concentration experiments to 2 classes by the concentration higher or lower than 24 h at 50% lethal concentration (LC50).

Then for each chemical we applied support vector machine (SVM) to do the 5-fold cross validation prediction tests only by the phenotypes quantified for 12 h experiments.

Results show that we can distinguish if the experiment’s concentration is higher or lower than the 24 h LC50 with a high degree of accuracy only by the quantified features from the time point of 12 h (Table 1). The classifier has an average accuracy of 91.2% by 5-fold cross-validation among all chemical experiments. These results indicate that we can assess the acute toxicity only based on the phenotypes quantified for 12-h experiments. This is a useful tool which will shorten the experimental observation time in the future.

**Table 1:** The cross validation prediction performance

Compound name	Cross-validation accuracy (%)	Compound name	Cross-validation accuracy (%)
Potassium chloride	93.3	Diquat dibromide	77.4
Cadmium chloride	78.6	Glycerol	96.3
Atropine sulfate	88.9	Sodium dichromate	90
Lactic acid	100	Manganese chloride	90.6
Anhydrous two propanol	98	Sodium chloride	82.5
Ethanol	90.6	Trichloroacetic acid	100
Ethylene glycol	94.6	Citric Acid	81.8
Sodium Fluoride	100	Orthoboric acid	98.2
Sodium Hypochlorite	89.6		

## Discussion

Large numbers of novel chemicals are produced annually and current models of toxicological studies are unable to meet the demands of modern scientific research and industry.

Studies utilizing lower order in vivo model organisms (such as *C. elegans*) have the opportunity to make a large contribution to the efficient risk assessment of chemicals toxicity. The development of better computational tools outlined in this article provides an opportunity for the creation of faster toxicity screening. Testing with the nematode *C. elegans* offers a unique approach that possesses advantages over cell-based in vitro assays, which are rapid but may not be physiologically relevant in the context of a whole organism. Mammalian studies, which may better represent human responses to toxic chemicals are slow and expensive and therefore the model we present could act as a useful complementary tool to mammalian trials [4].

Our model allows the growth of nematodes exposed to water soluble compounds of known toxicity to be assessed by a screening system in combination with automated phenotype profiling methods. Modeling results were used to rank order the test compounds according to their toxicity as determined by the nematode assay. This method proved useful in identifying very toxic compounds from compounds with moderate and low toxicity.

It is clear from our results that this new method of quantitative screening on 384 well plates for chemicals can detect the degree that toxicities alter the phenotypes of *C. elegans*. We found that phenotype profiling revealed conserved functions to classify and predict the toxicity of different chemicals using the nematode *C. elegans* as an in vivo model organism.

In this study, 19 standard chemicals acute toxicity tests based on the 384-well plate were conducted and the movie for each well was acquired automatically by digital camera (methods). About 100GB image data was processed by our program written in MATLAB to quantify 33 phenotypic features. From these 33 phenotypic features we performed Principle Component Analysis (PCA) to get several main components and classify them to several patterns according to 3 treatment times and several treatment concentrations, respectively. Results showed that the chemical toxicity could be predicted by the treatment concentration higher or lower than the 50% lethal concentration (LC50) at 24 h.

Our previous study [15] showed that the correlation of *C. elegans* LC50s (24 h) to rat LD50s is similar to the relationship between mouse and rat LD50s (*r* = 0.879), and was stronger than the correlation of NHK cell median inhibition concentrations (IC50)s vs. rat LD50s (*r* = 0.844). Here, the automatically quantified phenotypes show significant difference among different concentrations, it is similar to the previously study [15]. We also show that the automatically quantified phenotypes can be used to distinguish the chemical concentration is under/above the 24 h 50% lethal concentration (LC50 is determined by previous studies [15]).

In summary, the methodology presented using the rapid toxicity screening and evaluation system of non-rodents correlates with the traditional animal acute toxic effects and could be used to assess new chemicals or complex mixed chemicals. The scientific principle of replacement, reduction and refinement in regards to animal ethics is the central motivator of this research as it is hoped that these findings reduce the pressure currently on higher order animals to be relied upon for toxicology research. This study is significant improvement to current system methods which use *C. elegans* to pre-screen the toxicity of new chemicals. Our method can quantify a number of phenotypes that are difficult to calculate manually, such as the worm motility, worm size, width and gray intensity. When compared with traditional methods, our method delivers a more efficient and automated collection of data. This is possible mainly due to the advancements we have made in terms of automation, level of throughput and quality of data.

## Conclusions

This new method provides a powerful tool to researchers to understand the effects of chemical compounds. By combining a 384-well liquid toxic culturing plate with an automated image acquisition microscope system, we demonstrated quantitative screening of 19 chemical compounds using *C. elegans*. 33 worms’ features were identified for each chemical treatment. The results demonstrate the compounds with different toxicities were shown to alter the phenotypes of *C. elegans* in distinct and detectable patterns. Moreover, the power of phenotypic profiling in *C. elegans* revealed conserved functions to classify and predict the toxicity of different chemicals. The development of better computational tools outlined in this article provides an opportunity for the creation of faster toxicity screening in *C. elegans*; therefore, the model we present could act as a useful complementary tool to mammalian trials.

## Methods

### Chemicals and concentrations

The 19 standard chemicals used (at the third to sixth levels of the GHS) and their respective concentrations are listed in Additional file 1: Table S1. The four to eight gradient concentration levels were designed according to Horn method. All chemicals were obtained from Sigma-Aldrich Company, and diluted by K-medium (32 mM KCL and 52 mM NaCL in 1 L ultrapure water). We used K-medium as control to compare the alterations caused by the toxicants.

### Strains

All nematodes used were wild-type N2, originally obtained from the Caenorhabditis Genetics Center (CGC). Synchronized L1 worms were obtained by bleaching and then put on the nematode growth medium (NGM) plates seeded with *Escherichia coli* OP50 and kept at 20 °C. Worms at L4 stage were washed off the plate by K-medium and added into 384-well plates, each well containing ~ 20 worms. These synchronized, L4 stage worms were then ready for chemical treatment. Within 24 h of the chemical treatment experiment, we did not add bacteria to 384-well plates.

### Chemical treatment

Nineteen standard chemicals were administered at concentrations a third to sixth levels of the GHS for 384-well plate acute toxicity experiment tests. According to the *C. elegans* LC50 of chemicals, worms were exposed to chemicals at a single dosage of either; 10 mg/ml, 4.64 mg/ml, 2.15 mg/ml. 1 mg/ml, 0.464 mg/ml, 0.215 mg/ml, 0.1 mg/ml or 0.0464 mg/ml. Eight parallel tests were then carried out for the specific points of the concentration gradients for each chemical. Worms were then observed with each specific chemical condition in K-medium for 0, 12 h, or 24 h.

### Video capture

Images and video was captured at three distinct time points; 0, 12 and 24 h after noxious compounds were added. All images and videos were collected using a digital camera attached to a Nikon SMZ1000 automatic microscope.

### Image processing

**Fig. 5:**
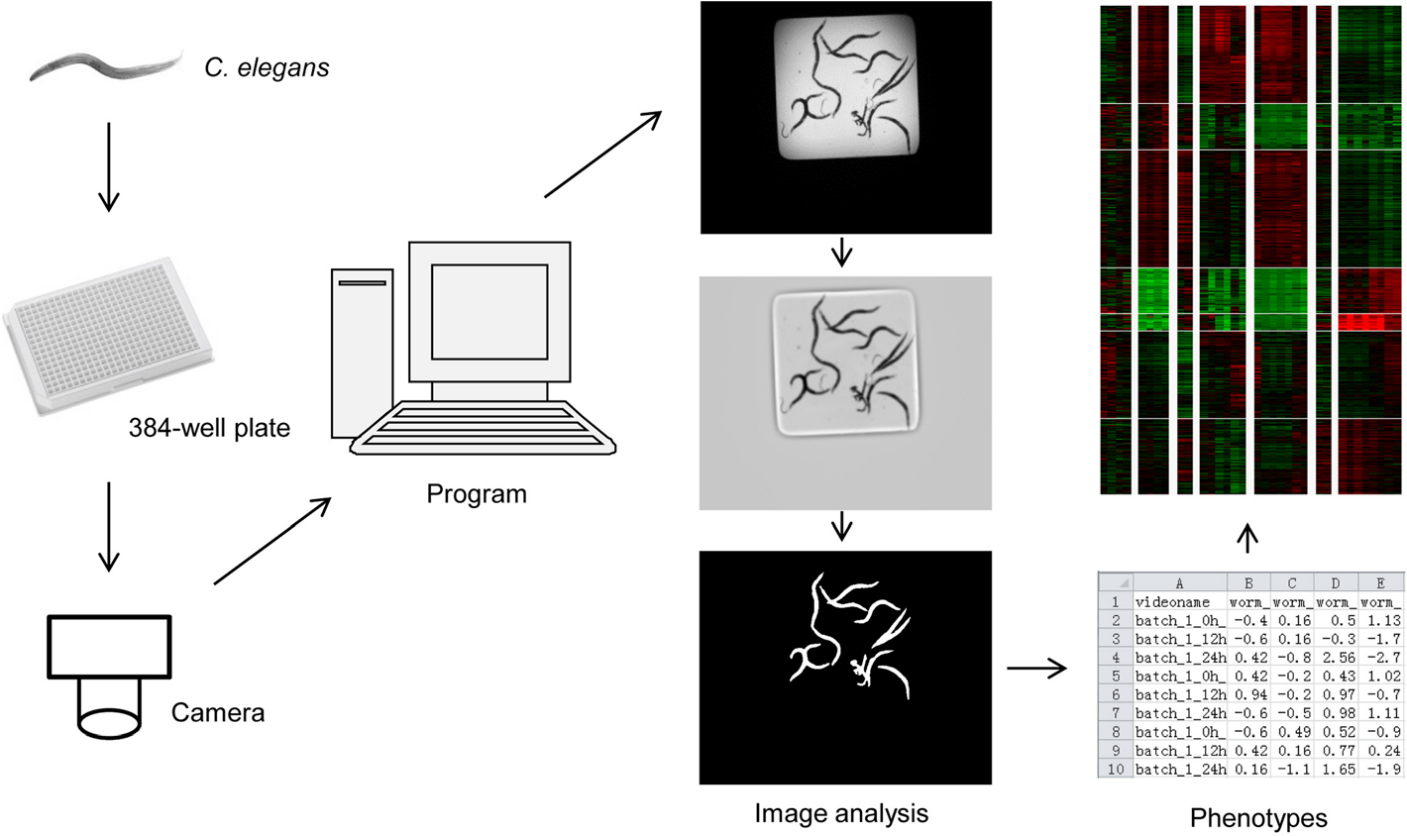
Method design. Mainly there are three steps in our method: (1) culture worms in 384-well plates with different chemicals and take video for each well; (2) process these videos, get each frame, reduce the uneven illumination, segment the image, and then quantify phenotypes for worms under each chemical treatment; (3) characterize different chemicals and their toxicities based on these quantified phenotypes

A program for image processing and analysis, automated worm recognition followed by automated features quantification was written using MATLAB software. The major procedures are described and shown in Fig. 5. After solving the uneven illumination problem [19], we were able to distinguish worms from the background of the well and quantify their features.

### Experimental setting

Following chemical treatments, we programmed the automated acquisition of video (7 frames per second for 2 s) for each well at three time points (0 h, 12 h and 24 h) by digital camera (methods). This resulted in a tremendous amount of image data (~100GB) automatically collected through all of the tests (3 plates of 384-well plate treatments). Next, we used the image processing method we developed using MATLAB to quantify valuable phenotypic features to ultimately analyze and to classify the toxicity effect on worms.

### Phenotypes definition and computation

Defined features include survival number related features, gray intensity related features, worm size, curvature, and motility related features:

- F1. The single worm number in this experiment;
- F2. The alive single worm number;
- F3. The worms’ disperse situation, which is computed by the ratio between single worm number and total worm number;
- F4. The average distance of all worms’ centroid;
- F5. The standard deviation of distance among all worms’ centroid;
- F6. The single worms’ average size;
- F7. The single worms’ average length;
- F8. The single worms’ average width;
- F9. The single worms’ average perimeter;
- F10. The single worms’ average gray intensity;
- F11. The single worms’ standard deviation of gray intensity;
- F12. The alive single worms’ average size;
- F13. The alive single worms’ average length;
- F14. The alive single worms’ average width;
- F15. The alive single worms’ average perimeter;
- F16. The alive single worms’ average gray intensity;
- F17. The alive single worms’ standard deviation of gray intensity;
- F18. The single worms’ major axis length, which is computed by the major axis of the ellipse that has the same normalized second central moments as the worm body region;
- F19. The alive single worms’ major axis length;
- F20. The single worms’ minor axis length, which is computed by the minor axis of the ellipse that has the same normalized second central moments as the worm body region;
- F21. The alive single worms’ minor axis length;
- F22. The ratio between single worms’ minor and major axis length, more close to 1 more close to one circle;
- F23. The ratio between alive single worms’ minor and major axis length;
- F24. The single worms’ eccentricity, which is the ratio of the distance between the foci of the ellipse and its major axis length, the value is between 0 and 1, an ellipse whose eccentricity is 0 is actually a circle, while an ellipse whose eccentricity is 1 is a line segment. It is computed from the outer ellipse of the worm’s body region. We used it to show the worm body bent degree, because lower eccentricity values indicate the worm would appear more bent;
- F25. The alive single worms’ eccentricity;
- F26. The single worms’ orientation, which is the angle between the x-axis and the major axis of the ellipse;
- F27. The alive single worms’ orientation;
- F28. The single worms’ motility, which is the moved area;
- F29. The single worms’ motility, which is computed by the moved area/worm size;
- F30. The alive single worms’ motility, which is the moved area;
- F31. The alive single worms’ motility, which is computed by the moved area/worm size;
- F32. The smoothed live worm number;
- F33. Survival rate.

### Statistical analysis

Results are presented as means ± SD. For the comparisons of phenotypes between each concentration treatment and control (k-medium) at each time point, we analyzed the data using unpaired two-sided Student’s *t*-Test in R. *P* values < 0.05 were considered statistically significant. The R packages were used for PCA [20] and SVM [21] analysis.

## Additional file

Additional file 1: **Figure S1**. Phenotypes of Diquat dibromide under different concentrations. **Figure S2**. Phenotypes of Sodium dichromate under different concentrations. **Table S1**. Experiments chemical concentration distribution. (PDF 297 kb)
