# Supplementary material for: Classification and prediction of toxicity of chemicals using an automated phenotypic profiling of Caenorhabditis elegans
Source: BMC Pharmacol Toxicol. 2018 Apr 18;19:18. doi: 10.1186/s40360-018-0208-3 (PMC5907177; doi:10.1186/s40360-018-0208-3)
Supplement: Supplementary file 1 — Figure S1. Phenotypes of Diquat dibromide under different concentrations. Figure S2. Phenotypes of Sodium dichromate under different concentrations. Table S1. Experiments chemical concentration distribution. (PDF 297 kb) [file 40360_2018_208_MOESM1_ESM.pdf]

Supplementary Material

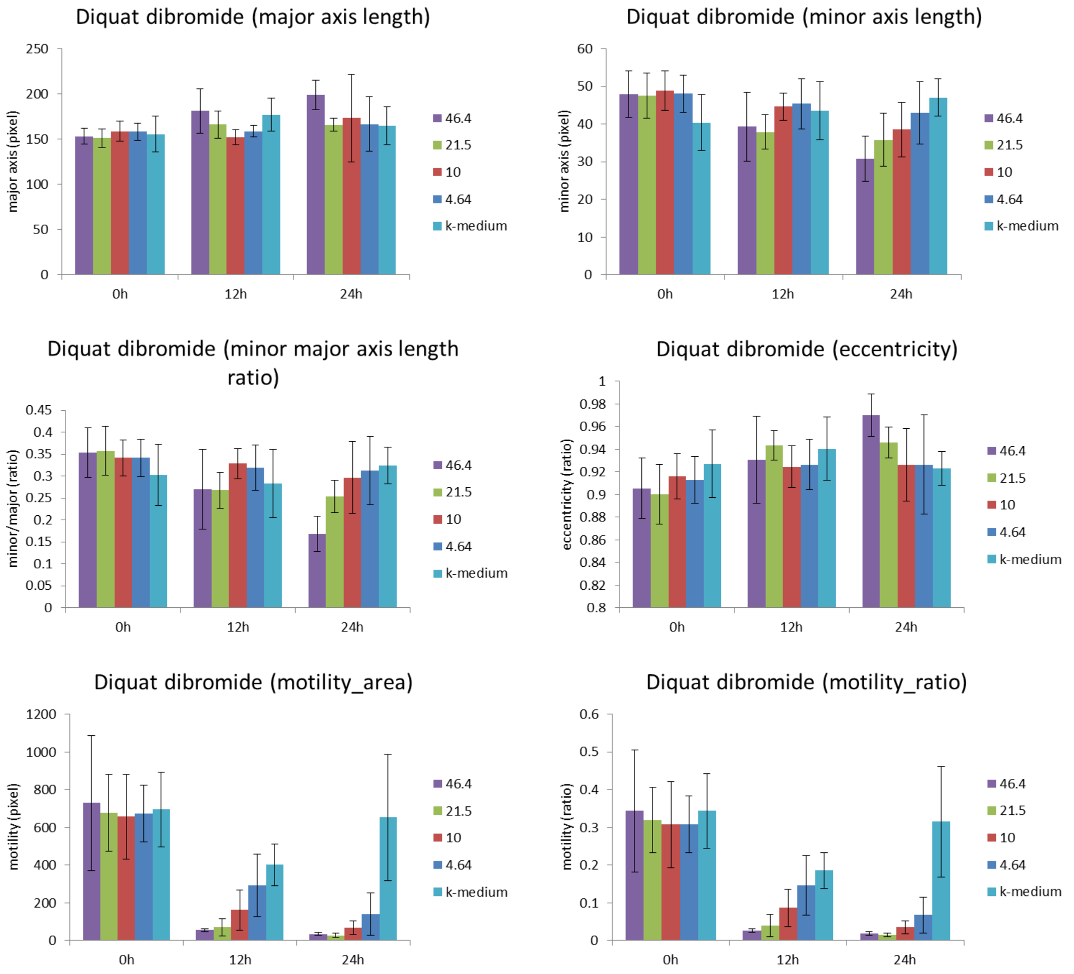

Fig. S1. Phenotypes of Diquat dibromide under different concentrations.

Bar plots shows the average quantification for each phenotype on single worms. Error bars denote +/- standard deviation (SD). Concentration unit: mg/ml.

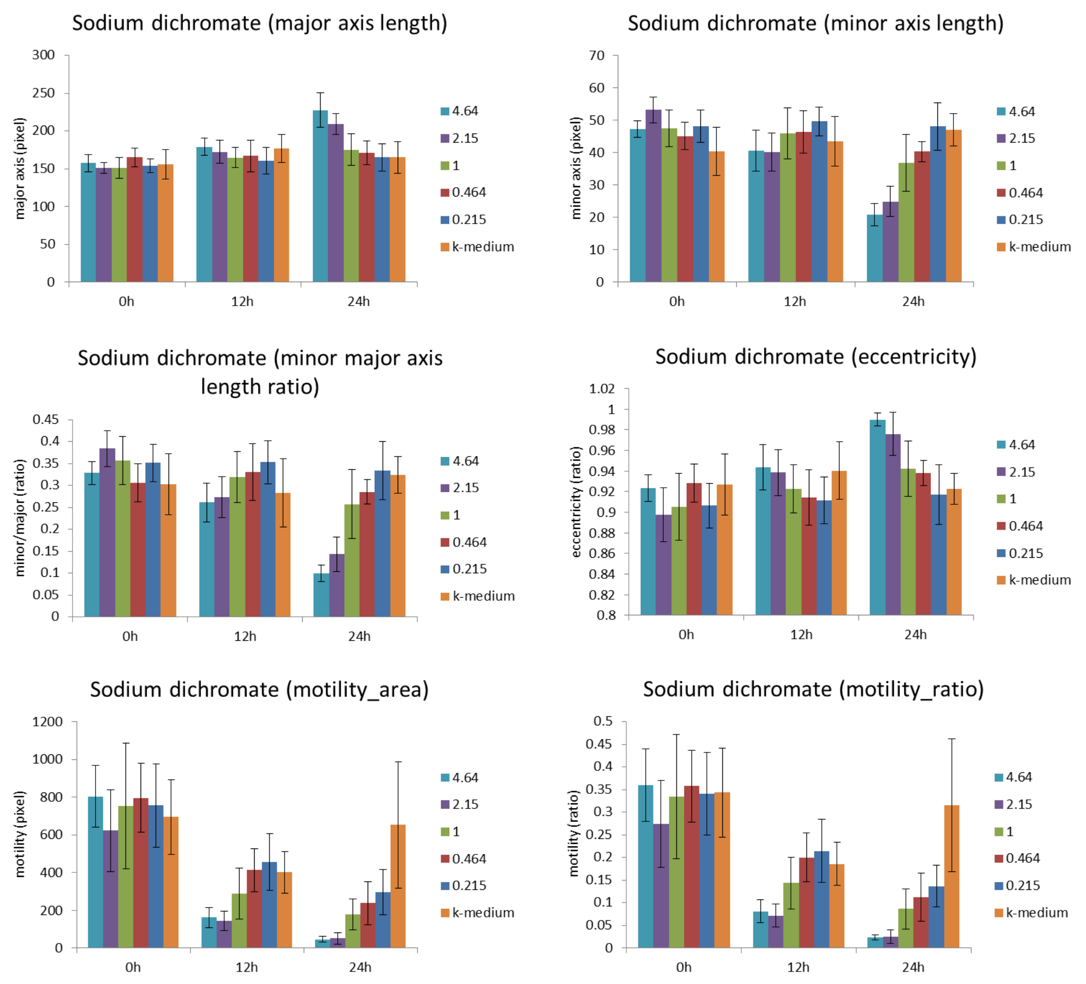

Fig. S2. Phenotypes of Sodium dichromate under different concentrations.

Bar plots shows the average quantification for each phenotype on single worms. Error bars denote +/- standard deviation (SD). Concentration unit: mg/ml.

Supplementary Table 1. Experiments chemical concentration distribution.

Compound name	Toxicity level	Dose (mg/ml)	*C. elegans* 24h LC50 (mg/ml)	Rat LD50 (mg/kg)	Mouse LD50 (mg/kg)
Cadmium chloride	III	0.0464<br>0.1<br>0.215<br>0.464<br>1<br>2.15<br>4.64<br>10	0.85	60	135
Sodium Fluoride	III	0.1<br>0.215<br>0.464<br>1<br>2.15<br>4.64	0.54	57	127
Sodium dichromate	III	0.215<br>0.464<br>1<br>2.15<br>4.64	0.59	180	51
Manganese chloride	III	4.64<br>10<br>21.5<br>46.4	12.29	1031	250
Diquat dibromide	III	4.64<br>10<br>21.5<br>46.4	13.6	233	160
Copper(II) sulfate pentahydrate	IV	0.01<br>0.0215<br>0.0464<br>0.1<br>0.215<br>0.464<br>1	0.00336	502	474
Atropine sulfate	IV	0.464<br>1<br>2.15<br>4.64<br>10<br>21.5<br>46.4	20.72	609.2	819

Potassium chloride	Ⅴ	2.15<br>4.64<br>10<br>21.5<br>46.4<br>100	24.62	1500	2799
Sodium chloride	Ⅴ	2.15<br>4.64<br>10<br>21.5<br>46.4<br>100	17.38	4000	4046
Lactic acid	Ⅴ	0.464<br>1<br>2.15<br>4.64<br>10	2.79	4875	3639
Orthoboric acid	Ⅴ	0.215<br>0.464<br>1<br>2.15<br>4.64<br>10<br>21.5	16.89	3450	3426
Ethanol	Ⅵ	2.15<br>4.64<br>10<br>21.5<br>46.4<br>100<br>215	52.62	3450	11324
Ethylene glycol	Ⅵ	4.64<br>10<br>21.5<br>46.4<br>100<br>215<br>464	124.51	5500	7161
Anhydrous two propanol	Ⅵ	2.15<br>4.64<br>10<br>21.5<br>46.4<br>100	24.47	3600	5105

		215			
Glycerol	Ⅵ	4.64<br>10<br>21.5<br>46.4<br>100<br>215<br>464	214.05	4090	19770
Trichloroacetic acid	Ⅵ	0.0464<br>0.1<br>0.215<br>0.464<br>1	0.44	4970	5229
Sodium Hypochlorite	Ⅵ	1<br>2.15<br>4.64<br>10<br>21.5<br>46.4	5.12	8910	8500
Citric Acid	Ⅵ	0.1<br>0.215<br>0.464<br>1<br>2.15<br>4.64<br>10	1.58	5040	5929
Gibberellic acid	Ⅵ	0.0215<br>0.0464<br>0.1<br>0.215<br>0.464<br>1	Unknown	6300	8500
